# On the Employment of Phosphate of Ammonia

**Published:** 1846-05

**Authors:** C. Voigt


*On the Employment of Phosphate of Ammonia.*

Prof. R. M. Huston:

In a trial made under my observation with this article, in the dose of only about three grains, a series of alarming and highly irritative symptoms was induced. In about an hour after its introduction into the stomach, the patient was attacked by a sense of tightness in the præcordia and around the chest; nausea; thirst; a hard, small, frequent pulse; and a collapsed state of the circulation. These disturbances were followed by fulness and tension in the head; heaviness in the limbs; and an unsteady tottering gait. The case seeming likely to become serious if unrelieved, was treated by a moderate bloodletting, a dose of senna and salts, and mucilaginous drink; which had the effect of affording relief, though some degree of gastric irritation remained for some hours subsequently. The first action of the medicine in this instance appeared to be violently irritating on the stomach. I had good reason to suppose that the preparation employed was pure.

Respectfully,

C. Voigt, M.D.

*March 30th*, 1846.

